# Cystic Fibrosis of the Pancreas: In Vitro Duct Models for CFTR-Targeted Translational Research

**DOI:** 10.3390/ijms27031279

**Published:** 2026-01-27

**Authors:** Alessandra Ludovico, Martina Battistini, Debora Baroni

**Affiliations:** Istituto di Biofisica, Consiglio Nazionale delle Ricerche (CNR), Via De Marini 6, 16149 Genova, Italy; ale.ludo89@gmail.com (A.L.); martinabattistini1@gmail.com (M.B.)

**Keywords:** cystic fibrosis (CF), CFTR, pancreatic duct, bicarbonate secretion (HCO_3_^−^), isolated primary pancreatic ducts, primary pancreatic ductal epithelial cells (PDECs), immortalized ductal cell lines, organoids (PDOs), organ-on-chip, Transwell/Snapwell

## Abstract

Cystic fibrosis (CF) is caused by loss-of-function variants in the cystic fibrosis transmembrane conductance regulator (CFTR) chloride and bicarbonate channel and affects multiple organs, with pancreatic involvement showing very high penetrance. In pancreatic ducts, CFTR drives secretion of alkaline, bicarbonate-rich fluid that maintains intraductal patency, neutralises gastric acid and permits safe delivery of digestive enzymes. Selective impairment of CFTR-dependent bicarbonate transport, even in the presence of residual chloride conductance, is strongly associated with exocrine pancreatic insufficiency, recurrent pancreatitis and cystic-fibrosis-related diabetes. These clinical manifestations are captured by pharmacodynamic anchors such as faecal elastase-1, steatorrhoea, pancreatitis burden and glycaemic control, providing clinically meaningful benchmarks for CFTR-targeted therapies. In this review, we summarise the principal mechanisms underlying pancreatic pathophysiology and the current approaches to clinical management. We then examine in vitro pancreatic duct models that are used to evaluate small molecules and emerging therapeutics targeting CFTR. These experimental systems include native tissue, primary cultures, organoids, co-cultures and microfluidic devices, each of which has its own advantages and limitations. Intact micro-perfused ducts provide the physiological benchmark for studying luminal pH control and bicarbonate (HCO_3_^−^) secretion. Primary pancreatic duct epithelial cells (PDECs) and pancreatic ductal organoids (PDO) preserve ductal identity, patient-specific genotype and key regulatory networks. Immortalised ductal cell lines grown on permeable supports enable scalable screening and structure activity analyses. Co-culture models and organ-on-chip devices incorporate inflammatory, stromal and endocrine components together with flow and shear and provide system-level readouts, including duct-islet communication. Across this complementary toolkit, we prioritise bicarbonate-relevant endpoints, including luminal and intracellular pH and direct measures of HCO_3_^−^ flux, to improve alignment between in vitro pharmacology and clinical pancreatic outcomes. The systematic use of complementary models should facilitate the discovery of next-generation CFTR modulators and adjunctive strategies with the greatest potential to protect both exocrine and endocrine pancreatic function in people with CF.

## 1. Introduction

Cystic fibrosis (CF) is the most common life-limiting autosomal recessive disease in Caucasian populations, with an incidence of approximately 1 in 2500–3500 live births depending on geography [1,2]. Newborn screening, optimised supportive care and CFTR modulators have markedly improved survival, and median life expectancy now exceeds 45 years in many countries [3]. Despite these advances, CF remains a multi-organ disease associated with substantial morbidity, repeated hospitalisations and a high lifelong treatment burden [4,5].

CF is caused by variants in the cystic fibrosis transmembrane conductance regulator (*CFTR*) gene, identified in 1989 [6]. *CFTR* encodes a 1480-amino-acid ATP-binding cassette (ABC) transporter that functions as a phosphorylation-activated anion channel. Two membrane-spanning domains form the ion-conducting pore, the two nucleotide-binding domains bind and hydrolyse ATP, and the regulatory domain is phosphorylated by protein kinase A [7]. These elements cooperate to regulate gating and permeation, allowing the transepithelial movement of chloride (Cl^−^) and bicarbonate (HCO_3_^−^) across secretory and absorptive epithelia, including airways, intestine, biliary tree, sweat ducts and pancreatic ducts [8]. In pancreatic ducts, CFTR-driven secretion of bicarbonate-rich fluid is essential to maintain ductal patency, neutralise gastric acid and support safe delivery of digestive enzymes to the duodenum [9].

More than 2000 *CFTR* variants have been reported, of which roughly 350 are disease-causing [10]. Mutations are grouped into six functional classes that reflect the underlying molecular defect: impaired protein synthesis (Class I), misfolding and trafficking (Class II), defective gating (Class III), reduced conductance (Class IV), reduced expression (Class V) and decreased stability at the plasma membrane (Class VI) [11,12]. This functional taxonomy maps directly onto pharmacological strategies, including potentiators for gating and conductance defects, correctors for misprocessing, stabilisers for unstable surface protein, read-through agents for nonsense variants and amplifiers for reduced expression [13,14,15,16]. F508del, a Class II processing mutation, is the most frequent variant worldwide and is present on at least one allele in the majority of European-ancestry patients [17].

Clinically, the lung is the main determinant of morbidity and mortality [18], but pancreatic disease has very high penetrance and is often among the earliest manifestations of CF [19,20,21,22,23,24]. In the pancreas, CFTR dysfunction causes acidic, viscous and protein-rich ductal secretions, intraductal obstruction and a cascade of inflammation, fibrosis and fatty replacement that culminates in exocrine pancreatic insufficiency (EPI) or recurrent pancreatitis, and ultimately, cystic-fibrosis-related diabetes (CFRD) [25,26,27]. These pancreatic outcomes are captured by measurable pharmacodynamic (PD) anchors such as faecal elastase-1, steatorrhoea, pancreatitis frequency and glycaemic control, and therefore provide clinically meaningful benchmarks for CFTR-targeted interventions [28].

From a translational perspective, not all CFTR rescue is equivalent at the organ level. For the pancreas, bicarbonate-related endpoints are particularly informative because selective deficits in HCO_3_^−^ conductance, even with preserved Cl^−^ transport, are associated with pancreatitis and pancreatic insufficiency [29,30]. Preclinical models should therefore quantify bicarbonate rescue explicitly rather than extrapolating from chloride-only measures.

This review examines pancreatic involvement in CF through a model-centric and assay-driven framework. We summarise key aspects of pancreatic pathophysiology and clinical management that define duct-relevant endpoints, then discuss in vitro pancreatic duct models used to evaluate CFTR-directed therapeutics. Particular attention is given to systems that preserve ductal identity and bicarbonate-dependent transport, including primary pancreatic ductal epithelial cells (PDECs), immortalised ductal lines, pancreatic ductal organoids (PDOs), co-culture models and organ-on-chip devices. Throughout, we highlight readouts relevant to CFTR pharmacology, including short-circuit current, ion flux, transepithelial electrical resistance (TEER) and transepithelial conductance (TEC), together with bicarbonate-focused endpoints such as luminal and intracellular pH, HCO_3_^−^ flux and regulatory pathways that shape anion selectivity. Because access to human tissue is limited and species regulation differs, the practical role of each model in translational studies is context dependent. What follows emphasises the strengths, limitations and realistic application of each model rather than a rigid discovery pipeline.

## 2. Pancreatic Disease in Cystic Fibrosis

The pancreas was central to the original description of CF, and the term “cystic fibrosis” refers to the fibrocystic lesions observed in exocrine tissue [31]. CFTR is highly expressed at the apical membrane of pancreatic ductal epithelial cells, where it coordinates chloride handling and bicarbonate secretion. In proximal ducts, bicarbonate is accumulated within the epithelium via basolateral transporters and secreted through CFTR into the lumen while chloride is reabsorbed. In more distal segments, where luminal chloride is low, CFTR predominantly functions as a bicarbonate channel and often operates in tight functional coupling with SLC26A6 and related exchangers [25,26,27,32,33,34].

Under physiological postprandial conditions, this machinery generates an alkaline, isotonic ductal fluid with bicarbonate concentrations approaching 140 mM. This fluid flushes zymogens through the ductal tree, neutralises gastric acid entering the duodenum and buffers protons co-released during acinar secretion, thereby limiting premature trypsinogen activation [35]. When CFTR activity is impaired, secretions become acidic, viscous and concentrated and volume is reduced. The combination of decreased HCO_3_^−^ content and altered rheology favours intraductal plugging, duct dilatation, focal acinar injury and progressive fibrosis [36,37,38]. Histopathological studies indicate that these changes can begin in utero, with lesions detectable in the second trimester, and eventually lead to extensive fatty replacement and loss of exocrine tissue, with only scattered islets and ducts remaining in end-stage glands [39].

Approximately 80 to 85% of individuals with severe *CFTR* mutations, particularly Classes I, II, III and VI, develop EPI and require lifelong pancreatic enzyme replacement therapy [29,30,40,41]. Around 15 to 20% remain pancreatic sufficient (PS), usually carrying milder Class IV or V variants, although some severe alleles can also underlie PS. Pancreatic sufficiency does not equate to normal pancreatic health. PS individuals frequently experience recurrent acute or chronic pancreatitis and may show biochemical evidence of exocrine injury despite preserved digestion. *CFTR* variants that selectively impair bicarbonate permeability while sparing chloride conductance are linked to idiopathic chronic pancreatitis and increased pancreatitis risk outside the CF population [29,30,40,41,42,43]. This emphasises the central role of bicarbonate transport in pancreatic homeostasis and reinforces the need for bicarbonate-aware assays in preclinical research.

Endocrine involvement is also clinically significant. CFRD develops in up to half of adults with CF and is often preceded by impaired glucose tolerance in adolescence [24,44,45,46]. CFRD combines features of type 1 and type 2 diabetes, with progressive loss of β-cell mass and function, variable insulin resistance and contributions from chronic inflammation and malnutrition. Histological studies show reduced islet mass, loss of insulin- and glucagon-producing cells, relative δ-cell enrichment and amyloid deposition. CFTR may directly modulate β-cell function in some contexts, although islet damage is also driven by surrounding exocrine fibrosis, local inflammation and altered microcirculation. Clinically, CFRD is associated with worse nutritional status, steeper decline in lung function and higher mortality than in CF patients without diabetes [44,45,46,47,48].

Pancreatic involvement interacts closely with lung disease [49,50]. EPI contributes to malnutrition, impaired immune function and reduced lung growth, while chronic pulmonary infection and inflammation increase energy expenditure and exacerbate nutritional deficits [51]. Better preservation of exocrine function correlates with improved pulmonary outcomes, and early optimisation of enzyme replacement and nutritional support supports more favourable trajectories for growth and lung function. CFRD further amplifies this vicious cycle by promoting catabolism and increasing glucose concentrations in airway surface liquid, which favours bacterial proliferation and weakens innate defence mechanisms [24,44,45,46,47,48].

From a translational perspective, several clinical biomarkers provide anchors that link cellular readouts to organ-level benefit. Faecal elastase-1 is a widely used marker of exocrine function [52,53]. Abdominal ultrasound and MRI detect structural changes including increased echogenicity, atrophy and cystic degeneration [54]. Serum immunoreactive trypsinogen reflects exocrine injury and is characteristically elevated in PS individuals [55]. Together, these biomarkers provide clinical comparators for in vitro endpoints and should guide calibration of pancreatic models developed for CFTR-targeted discovery.

## 3. Therapeutic Approaches to Pancreatic Disease in Cystic Fibrosis

### 3.1. Exocrine Pancreatic Insufficiency

Pancreatic enzyme replacement therapy (PERT) is the standard of care for individuals with EPI [56,57]. Enteric-coated enzyme microgranules are taken with meals and snacks, with doses tailored to dietary fat intake, symptoms and growth. Optimisation strategies include splitting doses during meals, checking adherence and storage and monitoring stool characteristics, weight, growth and fat-soluble vitamin status. Acid suppression can improve enzyme efficacy in selected patients by increasing duodenal pH and enhancing lipase activity. Nutritional management aims to compensate for increased energy expenditure and relies on high-calorie, high-fat diets with adequate protein and systematic supplementation of fat-soluble vitamins and micronutrients [56,57,58].

### 3.2. Pancreatic Inflammation and Pancreatitis

PS individuals with residual CFTR function have a higher risk of recurrent acute pancreatitis and may progress to chronic pancreatitis [59,60]. Standard supportive care remains central but CF-specific factors, such as impaired ductal bicarbonate secretion, viscous secretions and structural duct abnormalities, should be considered. Selected patients may benefit from endoscopic interventions when obstructive features are identified [61].

Emerging evidence suggests that CFTR modulators reduce pancreatitis frequency in responsive genotypes by improving ductal secretion. In contrast, pancreatitis has been reported after initiation of modulators in some previously EPI patients, possibly reflecting a transient imbalance between increased acinar enzyme output and still-limited ductal flow [62,63,64,65,66]. These observations support careful clinical monitoring during early modulator therapy in individuals with a history of pancreatitis.

### 3.3. Endocrine Involvement and CFRD

Screening for CFRD, typically by annual oral glucose tolerance testing from late childhood onwards, aims to detect dysglycaemia before overt clinical deterioration. Insulin is the treatment of choice once CFRD is diagnosed and early initiation improves nutritional status, lung function and survival [67]. Management integrates diabetes education, nutrition aligned with CF dietary goals and surveillance for microvascular complications, while avoiding restrictive regimens that may worsen undernutrition. Continuous glucose monitoring can identify early abnormalities and refine treatment strategies [24,44,45,46,47,48,68,69].

### 3.4. CFTR Modulators and Pancreatic Outcomes

CFTR modulators are beginning to alter the natural history of pancreatic disease, particularly when initiated early and in highly responsive genotypes. In infants and young children with gating mutations, ivacaftor increases faecal elastase-1, improves growth and reduces steatorrhoea, and a proportion of treated children transition from severe EPI to pancreatic-sufficient ranges, especially when treatment begins early [67,70]. Observational programmes report broader gastrointestinal benefits consistent with enhanced CFTR activity along the gut-pancreas axis [71]. Modulators appear to reduce pancreatitis incidence in PS individuals with gating variants, whereas transient pancreatitis has been described after ivacaftor or ETI initiation in some EPI patients [72,73,74,75,76]. These findings support a genotype- and age-dependent window of pancreatic plasticity. Adults with long-standing EPI rarely recover full exocrine function, whereas children treated early show a higher likelihood of partial restoration.

Data on endocrine endpoints remain heterogeneous, with reports of reduced insulin requirements or improved glucose tolerance, although systematic pancreas-focused trials are still needed. A case report describes a 48-year-old patient with a gating mutation who experienced a marked reduction in recurrent pancreatitis and an increase in faecal elastase-1 after ivacaftor, illustrating that meaningful exocrine recovery may occur even in adulthood [77,78,79].

## 4. In Vitro Models of Pancreatic Ducts as Experimental Systems for CFTR-Directed Studies

Pancreatic duct models comprise a complementary set of experimental systems that can be used to investigate CFTR-dependent chloride and bicarbonate transport with varying degrees of physiological relevance, scalability and experimental control. Rather than being used in isolation, these models are typically applied in combination: scalable immortalised cell lines support early mechanistic interrogation, patient-derived primary cells and organoids enable confirmation under bicarbonate-aware conditions, and higher-complexity co-culture or microfluidic systems allow evaluation under flow, shear and paracrine cues. Intact micro-perfused ducts remain the physiological reference for bicarbonate-rich secretion.

Throughout this section, the term “experimental model” refers to the biological preparation (e.g., primary ducts, organoids or immortalised ductal lines), whereas “assay” or “readout” refer to the specific measurement performed (e.g., ΔIsc, luminal or intracellular pH, or forskolin-induced swelling). Model–readout combinations are specified only when explicitly coupled.

### 4.1. Intact Micro-Dissected Interlobular Ducts

Intact interlobular pancreatic ducts provide the closest ex vivo approximation of native ductal physiology. These small duct segments preserve epithelial architecture, lumen, polarity and basement membrane, allowing real-time assessment of luminal pH regulation, fluid movement and bicarbonate-rich secretion under controlled stimulation. Owing to their fidelity to the in vivo state, micro-dissected ducts are widely considered the physiological benchmark for evaluating pancreatic CFTR function and bicarbonate-centric transport.

**Tissue origin and isolation.** Interlobular ducts are typically isolated from rodents or from larger species, such as pigs or ferrets, which model aspects of human ductal physiology and exocrine disease with higher fidelity. A fresh pancreas is placed in ice-cold, oxygenated buffer and lobules are carefully teased apart under a stereomicroscope. Interlobular ducts are identified by morphology, freed from surrounding acinar tissue and transferred to a perfusion chamber for cannulation. Cannulated ducts are mounted in a temperature-regulated micro-perfusion chamber and independently perfused luminally and basolaterally with defined, oxygenated, CO_2_/HCO_3_^−^-buffered solutions. Continuous gas equilibration and strict temperature control are essential for stable pH and reproducible secretory signals [80,81,82,83,84,85,86,87].

**Stimulatory protocols and specific functional readouts.** Luminal pH is monitored using ratiometric fluorescent dyes calibrated in situ or by high-impedance microelectrodes positioned within the lumen, enabling high temporal resolution of bicarbonate-dependent alkalinization [88,89,90]. Fluid secretion is quantified by video microscopy from time-resolved changes in luminal diameter or volume after geometric calibration [91,92,93]. Bicarbonate flux is inferred by integrating luminal pH with secretion rate using Henderson–Hasselbalch relationships and established buffer parameters [92,93,94,95,96,97].

Forskolin (with or without IBMX) activates cAMP–PKA signalling, while secretin provides a physiological agonist of the same pathway. ATP or carbachol engage Ca^2+^-dependent secretion and reveal functional coupling between CFTR and SLC26 exchangers. CFTR specificity is confirmed using luminal CFTRinh-172, whereas basolateral bumetanide limits NKCC1-mediated chloride loading. DIDS or H_2_DIDS can interrogate SLC26-mediated exchange, and low-chloride perfusates or targeted modulation of WNK–SPAK signalling enable the analysis of CFTR anion selectivity towards bicarbonate. Complementary measurements, including intracellular pH, mitochondrial membrane potential and NAD(P)H autofluorescence, can link secretory activity to epithelial metabolism [98,99,100].

**Utility for CFTR-directed studies**. Because intact micro-perfused ducts preserve native polarity, luminal access and physiological ion gradients, they provide the highest-fidelity ex vivo platform for evaluating CFTR-directed therapies. In cerulein-induced pancreatitis, CFTR mRNA expression is preserved or increased, yet the protein becomes progressively mislocalised along the ductal epithelium, coinciding with disturbed bicarbonate transport. Pre-treatment with tezacaftor (VX-661) and ivacaftor (VX-770) partially restores CFTR function, enhances bicarbonate-dependent fluid secretion in isolated ducts and reduces pancreatic tissue damage by approximately 20–30% in vivo. Complementary evidence from autoimmune models supports a causal role for ductal CFTR activity in maintaining exocrine homeostasis, linking restoration of bicarbonate-rich secretion to improved acinar physiology and reduced inflammation [101,102].

**Strengths, limitations and best practice.** Strengths include preservation of native polarity and direct quantification of luminal pH and bicarbonate-rich secretion. Limitations include low throughput, technical complexity and species-specific regulatory differences. Best practice includes rapid cold handling, continuous oxygenation, precise temperature regulation, calibration of pH sensors and standardised stimulation and inhibition protocols.

**Role in translational studies**. Micro-perfused ducts serve as a high-fidelity physiological reference to verify bicarbonate-rich secretion under defined stimuli and inhibitors. Given low throughput and technical demands, their most realistic value is as a benchmark to calibrate and qualify readouts from simpler epithelial systems.

### 4.2. Primary Pancreatic Ductal Epithelial Cells (PDECs) in Culture

Primary PDECs provide a reductionist yet physiologically anchored model of duct epithelium. When expanded as polarised monolayers on permeable supports, they preserve apical-basal polarity and key regulatory coupling that supports chloride and bicarbonate secretion [101,102,103,104,105,106,107,108,109,110].

**Tissue origin and isolation.** Human PDECs are typically obtained from surgical resections, donor organs or limited tissue and enriched for ductal identity (for example, KRT19^+^ and SOX9^+^) [111]. Contemporary protocols often include depletion of stromal contaminants (for example, CD90^+^) to limit fibroblast overgrowth. Rodent PDECs can be isolated using lectin-based enrichment or sorting strategies [112,113]. Ferret and pig PDECs offer high translational relevance [114,115,116].

**Figure 1:**
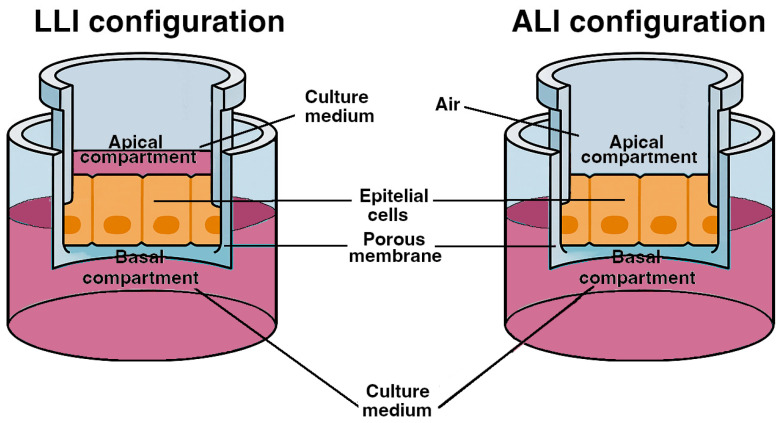
Transwell and Snapwell inserts are widely used in vitro systems for generating polarised pancreatic duct epithelial monolayers from immortalised lines or PDECs. On collagen IV or laminin-coated membranes, these cells form tight junctions with high transepithelial resistance and apical enrichment of CFTR. Transwell inserts enable optical assays, including intracellular and apical pH imaging and halide flux, and allow TEER and TEC measurements, enabling parallel evaluation of Cl^−^ and HCO_3_^−^ transport under controlled conditions. Snapwell inserts are optimal for classical Ussing-chamber recordings because the detachable ring format ensures stable voltage-clamp conditions and quantitative ΔIsc responses suitable for dose–response analyses. The left panel illustrates liquid–liquid interface (LLI) culture, with medium in both apical and basolateral compartments, supporting expansion and routine barrier monitoring. The right panel shows air–liquid interface (ALI) culture, where apical medium is removed after initial expansion and nutrition is supplied from the basolateral side. ALI conditions can enhance epithelial differentiation and apical accessibility for live imaging and acute CFTR potentiation. Perfused insert configurations introduce controlled low-shear flow and provide a conceptual bridge toward microfluidic organ-on-chip systems.

When seeded onto collagen IV- or laminin-coated inserts (Figure 1), PDECs form cohesive monolayers with tight junctions and apical enrichment of CFTR. Barrier formation is quantified by TEER and TEC, and polarity enables independent apical and basolateral stimulation [117,118].

**Stimulatory protocols and specific functional readouts.** Ussing-chamber electrophysiology quantifies CFTR-dependent transport as changes in short-circuit current. cAMP activation is achieved with forskolin (with or without IBMX) or secretin. ATP or carbachol probes Ca^2+^-dependent secretion and exchanger coupling. CFTR specificity is assessed using CFTRinh-172 or glybenclamide. Intracellular or apical pH can be monitored using fluorescent indicators such as BCECF, and halide-flux assays provide complementary conductance measures.

**Utility for CFTR-directed studies.** Ussing-chamber electrophysiology quantifies CFTR-dependent transport as changes in short-circuit current. cAMP activation is achieved with forskolin (with or without IBMX) or secretin. ATP or carbachol probes Ca^2+^-dependent secretion and exchanger coupling. CFTR specificity is assessed using CFTRinh-172 or glybenclamide. Intracellular or apical pH can be monitored using fluorescent indicators such as BCECF, and halide-flux assays provide complementary conductance measures [119,120,121,122,123].

**Strengths, limitations and best practice.** Strengths include physiological relevance and retention of duct-specific regulatory circuits. Limitations include restricted tissue availability, finite lifespan and variability across donors and passages. Best practice includes careful epithelial enrichment, early verification of duct markers, routine functional testing and explicit reporting of genotype, buffer composition and acute versus chronic treatment timing.

**Role in translational studies.** Primary monolayers are best suited for confirmation and mechanistic dissection under bicarbonate-aware conditions, especially when donor genotype and clinical phenotype are known. They complement, rather than replace, scalable immortalised lines and can precede higher-complexity settings such as co-cultures or chips.

### 4.3. Immortalised Pancreatic Ductal Epithelial Cell Lines and Epithelial Assay Models

Immortalised pancreatic ductal epithelial cell lines offer a scalable, reproducible system to study epithelial polarity, CFTR-dependent ion transport and inflammatory outputs. When cultured on permeable supports, they enable quantitative assessment of chloride and bicarbonate-related endpoints and provide the initial screening tier before confirmation in PDO-derived monolayers, primary PDECs or intact ducts [124,125].

**Cellular origin and representative lines.** CFPAC-1, derived from a pancreatic adenocarcinoma in a patient with CF, carries endogenous F508del-CFTR and remains a canonical CF ductal model for transepithelial transport studies [126,127]. CAPAN-1 expresses wild-type CFTR and is often used as a non-CF reference [128]. HPDE and HPDE6-E6E7 preserve near-normal ductal features and can support the generation of isogenic CFTR variants through targeted engineering [129]. Additional PDAC-derived lines such as MIA PaCa-2, BxPC-3 and AsPC-1 are widely used for inflammatory signalling and tumour–duct interactions, but their quantitative utility for CFTR transport is more limited [130,131,132].

**Stimulatory protocols and specific functional readouts.** Short-circuit current recordings provide sensitive measurements of CFTR-dependent chloride and bicarbonate transport, while TEER/TEC report barrier integrity. Intracellular and apical pH imaging, often under low-chloride gradients, helps resolve bicarbonate conductance and CFTR-SLC26 coupling [133]. Halide-flux assays provide orthogonal conductance readouts. These models also support profiling of cytokines and signalling pathways relevant to inflammation [134,135,136]. Stimulation uses forskolin with or without IBMX, secretin and Ca^2+^-dependent agonists, with CFTR inhibition as a specificity control.

**Utility for CFTR-directed studies**. Immortalised monolayers (Figure 1) are well-suited for defining dose–response relationships and distinguishing acute potentiation from chronic correction. In CFPAC-1, chronic corrector treatment followed by acute potentiation produces CFTRinh-172-sensitive ΔIsc increases and improves pH-related endpoints. CAPAN-1 and HPDE-derived systems allow isogenic comparisons and quantification of bicarbonate-relevant outputs, including apical alkalinisation and HCO_3_^−^-dependent ΔIsc. [137,138].

**Strengths, limitations and best practice.** Strengths include scalability, low cost and ease of genetic manipulation. Limitations include oncogenic background, passage-dependent drift and incomplete recapitulation of flow-dependent duct physiology. Best practice includes verification of epithelial markers, stability of CFTR function across passages and careful reporting of insert properties, coating, buffer composition and timing of pharmacological interventions.

**Role in translational studies.** Immortalised monolayers remain the practical workhorse for SAR and mechanism, provided that chloride-based improvements are corroborated by bicarbonate-relevant readouts and subsequently verified in primary or PDO-derived epithelia.

### 4.4. Pancreatic Ductal Organoids (3D)

Pancreatic ductal organoids (PDOs) recapitulate a self-organising, apico-basally polarised ductal epithelium that preserves endogenous CFTR expression and the regulatory networks governing chloride and bicarbonate secretion. When expanded long-term in Wnt- and R-spondin-enriched media, they maintain stable growth, sustained epithelial polarity and transport competence, closely reflecting the physiology of the native pancreatic duct and complementing 2D systems [139,140].

**Figure 2:**
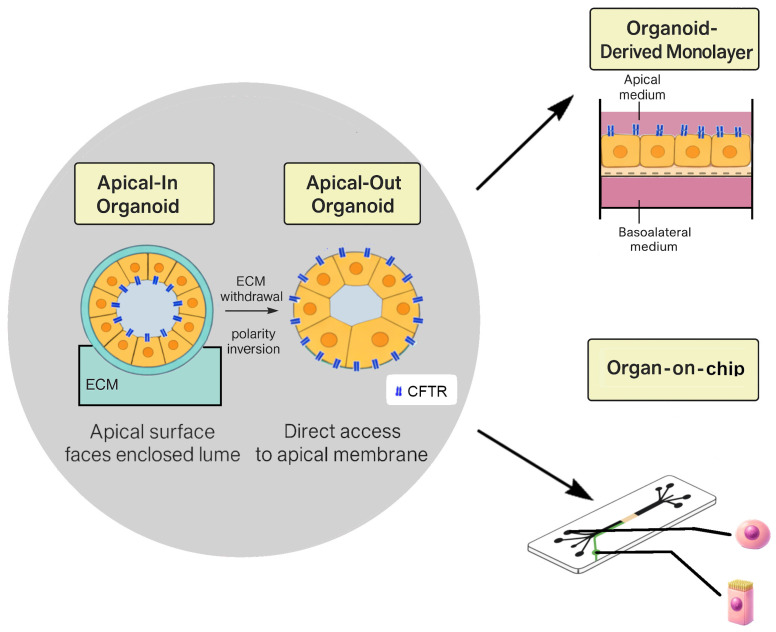
PDOs can be generated with either apical-in or apical-out polarity depending on extracellular matrix conditions. In the default apical-in configuration, the CFTR-rich apical membrane faces the enclosed lumen. Short-term ECM withdrawal induces polarity inversion and produces apical-out organoids in which the apical surface is exposed to the external medium, facilitating direct access for functional assays. Organoids can be dissociated into small clusters and seeded onto permeable supports to generate planar organoid-derived monolayers with measurable TEER and compatibility with Ussing-type electrophysiology. These epithelial formats can be integrated into microfluidic organ-on-chip devices, where they can also be combined with stromal, immune or endocrine partners to create co-culture models that enable physiologically relevant duct–microenvironment interactions and quantitative CFTR pharmacology under controlled flow conditions.

Before detailing tissue sources, it is useful to distinguish PDO formats that differ in polarity and assay accessibility. Standard organoids are typically apical-in, with the CFTR-rich apical surface facing an enclosed lumen, which may require microinjection for direct luminal access. Short-term extracellular matrix modulation can induce polarity inversion and generate apical-out organoids, exposing the apical membrane to the external medium and facilitating functional measurements without microinjection [141,142]. Organoids can also be converted into planar organoid-derived monolayers on permeable supports, enabling TEER measurements and Ussing-type electrophysiology (Figure 2).

**Cellular origin and representative lines.** PDOs derive exclusively from primary pancreatic tissue and cannot be generated from established pancreatic cell lines. Human or murine organoids are most commonly produced from micro-dissected ductal fragments obtained from donor pancreas, surgical resections or individuals with CF. Following mechanical and enzymatic dissociation, epithelial fragments are embedded in Matrigel and cultured in media supplemented with EGF, Noggin, R-spondin, Wnt3a, FGF10 and inhibitors of BMP and TGF-β signalling. These conditions support the formation of cystic organoids with intact luminal architecture and coordinated polarity [141,143,144].

Direct isolation yields ductal epithelia expressing KRT7, KRT8, KRT19, CFTR and ZO-1 and lacks acinar or endocrine markers [145]. Acinar cells can also generate duct-like organoids via acinar-to-ductal metaplasia in Wnt-rich conditions, although transcriptional memory may persist [146]. Across preparations, contaminants are progressively lost with passaging, and ductal signatures become dominant as cultures stabilise.

**Stimulatory protocols and specific functional readouts.** PDOs preserve regulatory wiring, controlling ductal secretion. Assays include forskolin-induced swelling, intracellular or apical pH imaging, short-circuit current in organoid-derived monolayers, halide-flux assays and analysis of CFTR-exchanger coupling and regulatory signalling. Recent evidence indicates that swelling in pancreatic PDOs is predominantly chloride-dependent and only partially sensitive to bicarbonate depletion, supporting the use of swelling as a CFTR-sensitive endpoint while highlighting the need to include bicarbonate-specific readouts for a complete functional profile [147,148,149,150,151].

**Utility for CFTR-directed studies**. PDOs reproduce CFTR-dependent secretory phenotypes and support genotype-specific profiling. Pancreatic organoids generated from individuals with CF recapitulate defective CFTR-dependent secretion, enabling testing of mutation-specific rescue strategies, including modulators and emerging nucleic-acid-based approaches. Compared with intestinal organoids, systematic evaluation of clinically approved modulators in pancreatic PDOs remains less extensive, but the preservation of patient genotype and duct-relevant transport mechanisms position PDOs as a valuable platform for precision pharmacology.

**Strengths, limitations and best practice**. PDOs offer patient anchoring, preserved polarity, compatibility with medium-throughput pharmacology and access to bicarbonate-centric endpoints that are incompletely resolved in many immortalised systems. Limitations include extracellular matrix (ECM) variability and, in apical-in cysts, restricted access to the luminal membrane without polarity inversion or microinjection. Best practice includes reporting genotype, passage, ECM formulation and batch, polarity state, buffer composition and detailed stimulation and inhibition protocols, distinguishing acute potentiation from chronic correction and providing both chloride- and bicarbonate-relevant effect sizes.

Role in translational studies. PDOs and PDO-derived monolayers support genotype-anchored confirmation and theratyping. Their value lies in linking epithelial pharmacology to duct-relevant bicarbonate transport, acknowledging matrix variability and the need for polarity control.

### 4.5. Co-Colture and Organ-on-Chip Systems of Pancreatic Duct Epithelium (2D and 3D)

Co-culture systems extend pancreatic duct models beyond epithelial monocultures by reintroducing cellular interactions that shape duct physiology and pathophysiology in vivo. Building on polarity-controlled organoid formats, organoid-derived monolayers and microfluidic epithelial devices, these systems allow integration of stromal, immune or endocrine partners while preserving CFTR-dependent transport readouts. Despite pancreas-specific co-culture evidence outside oncology and specialised chip studies remaining comparatively limited, the underlying architectures and CFTR-sensitive assays are transferable to non-malignant ductal systems when configured for bicarbonate-dependent physiology (Figure 2).

**Cellular origin and representative composing cell types**. Ductal epithelia are typically provided by primary PDECs, PDO-derived monolayers or, in higher-throughput settings, immortalised ductal lines. Partner cell types are selected according to the biological question. Macrophages are commonly incorporated to model inflammatory stress and cytokine-driven remodelling, and organoid-based co-culture and chip approaches have been highlighted as enabling technologies for immune-epithelial interaction studies [152,153]. Mesenchymal or stellate-like cells enable interrogation of fibro-inflammatory signalling, whereas endothelial cells become relevant in perfused configurations that introduce barrier cues and shear-related inputs. Integration of pancreatic islets enables direct testing of endocrine–exocrine coupling, particularly in experimental models that assess whether ductal transport defects propagate to β-cell dysfunction [154].

**Co-culture architecture and platform design.** In 2D, Transwell or Snapwell inserts preserve apical-basal polarity and allow immune or stromal cells to be positioned on either side of the epithelial barrier, enabling controlled modelling of luminal challenge or basolateral inflammatory signalling. In 3D, organoids embedded in ECM typically maintain apical-in polarity, while polarity-reversal strategies generate apical-out configurations that provide direct access to the CFTR-rich membrane. Microfluidic organ-on-chip devices extend these concepts by introducing controlled perfusion, shear stress and stable chemical gradients, enabling longitudinal stimulation and monitoring under defined flow conditions [155].

**Functional readouts and bicarbonate-aware conditions**. For pancreatic applications, the experimental configuration should preserve bicarbonate-dependent physiology. CO_2_/HCO_3_^−^-equilibrated buffers are preferred over HEPES-based media when pH regulation and bicarbonate flux are primary outcomes. Stimulation paradigms typically engage cAMP–PKA pathways (forskolin, IBMX, secretin), while carbachol probes Ca^2+^-dependent secretion. Inflammatory challenges (for example IL-1β, TNF-α, or LPS) are used to interrogate immune–epithelial crosstalk. Readouts include electrophysiology, TEER, intracellular and apical pH measurements, halide-flux surrogates, cytokine profiling and mucin secretion. In duct–islet configurations, glucose-stimulated insulin secretion provides a direct endocrine output that links ductal physiology to β-cell function [155,156].

**Utility for CFTR-directed studies**. Co-culture and chip-based devices provide an intermediate level of complexity between epithelial monocultures and intact duct micro-perfusion. Patient-derived pancreas-on-a-chip systems have been used to model CF-related pancreatic disorders and to test whether restoring ductal CFTR function rescues multicompartment phenotypes under controlled flow [156]. More broadly, these experimental systems enable evaluation of whether CFTR potentiation or correction remains effective under inflammatory or stromal stress. Methodologies developed in pancreatic cancer PDO co-culture studies provide practical frameworks for immune-epithelial interrogation and can be adapted to non-malignant ductal systems when assay conditions are optimised for ductal transport rather than tumour biology [152,153,154,155,156].

**Strengths, limitations and best practice**. Strengths include controlled cell ratios and spatial organisation, preservation of epithelial polarity and access to multicellular endpoints. Chips add perfusion and shear, supporting longitudinal assessment under near-physiological stimulation. Limitations include increased complexity, reduced throughput, phenotypic drift in partner cells and standardisation challenges. Best practice includes explicit reporting of epithelial provenance and passage, validation of polarity and ductal markers, documentation of partner-cell identity, matrix composition, device architecture and buffer formulation, together with CFTR-specific pharmacological controls and bicarbonate-sensitive endpoints.

**Role in translational studies.** Co-culture and chip devices are suited to stress-testing epithelial rescue under inflammatory, stromal, endocrine and flow constraints. They provide system-level insight but are not intended for primary screening. Taken together, these considerations suggest that no single in vitro pancreatic duct model can fully capture the complexity of cystic-fibrosis-related pancreatic disease. Instead, each system addresses different experimental and translational questions, so it is important to select the most appropriate model for the specific research objective. To support informed model selection, Table 1 provides a comparative overview of the main in vitro pancreatic duct models, summarising their respective strengths, limitations and areas of applicability.

Immortalised epithelial cell lines are robust and scalable for early mechanistic interrogation but provide limited tissue fidelity. Patient-derived organoids and organoid-derived monolayers improve physiological relevance and enable genotype-specific analyses, albeit at the cost of reduced throughput and increased variability. Co-culture and organ-on-chip platforms introduce multicellular interactions and flow and stress conditions that enable the validation of CFTR rescue under inflammatory, stromal or endocrine constraints. However, they remain technically demanding. Finally, ex vivo micro-perfused pancreatic ducts most closely approximate native duct physiology, primarily serving as a confirmatory reference for bicarbonate-dependent secretion rather than as a discovery tool.

## 5. Conclusions and Perspectives

Pancreatic disease in CF is driven primarily by loss of CFTR-dependent HCO_3_^−^ secretion, with consequences including exocrine insufficiency, pancreatitis and CFRD. This pathophysiological axis provides a practical framework for translational research. In vitro models should be selected and interpreted according to how directly they report bicarbonate-rich secretion and how clearly they can be aligned with clinical endpoints such as faecal elastase-1, pancreatitis burden and glycaemic control.

Taken together, intact micro-perfused ducts, primary PDECs, immortalised lines, PDOs, co-culture systems and organ-on-chip devices form a complementary toolkit. Immortalised monolayers support early screening and structure-activity work. PDO-derived epithelia and primary PDECs anchor findings in duct-relevant physiology and genotype specificity. Co-culture and chip systems add flow, shear and multicellular context, and ex vivo ducts provide the physiological reference for bicarbonate-rich secretion. In practice, most pancreas-focused programmes combine scalable epithelial systems for SAR with bicarbonate-aware confirmation in patient-anchored models, reserving intact ducts as a physiological reference.

Looking forward, the use of these in vitro models can be extended beyond the study of the effect of current corrector–potentiator regimens. Gene editing, mRNA replacement, antisense oligonucleotides, stabilisers, amplifiers, read-through agents and strategies that bias CFTR toward greater bicarbonate conductance will require evaluation in pancreas-appropriate systems using explicit bicarbonate-centric readouts, not only chloride currents. Progress will depend on rigorous reporting and sharing of protocols, including buffer composition, polarity markers, insert and device characteristics and statistical treatment, enabling integration across laboratories. If these conditions are met, bicarbonate-aware pancreatic models should help close the translational gap between epithelial CFTR rescue and durable organ-level protection of both exocrine and endocrine function in people with CF.

## Figures and Tables

**Table 1:** Comparative overview of the main in vitro pancreatic duct models used to study CFTR-directed therapeutic strategies. The table highlights key strengths, limitations and the types of experimental or translational questions for which each model is most appropriately suited.

Experimental Model	Strengths	Limitations	Questions Addressed
Interlobular ducts	Native architecture; direct assessment of bicarbonate-rich secretion	Very limited availability; low throughput; short experimental window	Physiological validation; benchmarking of epithelial models
PDECs	Preserved epithelial polarity; physiologically regulatory coupling; bicarbonate-aware transport	Limited tissue access; finite lifespan; donor variability	Mechanistic confirmation; genotype-informed functional analysis
Pancreatic duct epithelial cell lines	Reproducible; scalable; compatible with quantitative CFTR assays	Limited physiological relevance; oncogenic background in some lines	Early mechanistic studies; dose–response analysis; acute versus chronic modulation assessment
PDO and PDO-derived monolayers	High physiological relevance; preservation of patient genotype; multiple functional readouts	ECM variability; limited throughput; polarity-dependent accessibility	Genotype-specific profiling; precision pharmacology; bicarbonate-relevant validation
Organ-on-chip systems	Controlled flow and gradients; dynamic mechanical cues	Technical complexity; limited accessibility; specialised infrastructure	System-level validation under defined flow and shear conditions
Co-culture systems	Inclusion of immune, stromal or endocrine components	Increased complexity; reduced standardisation; lower throughput	CFTR rescue under inflammatory, stromal or paracrine stress

## Data Availability

No new data were created or analyzed in this study. Data sharing is not applicable to this article.
